# Targeting β-catenin overcomes MEK inhibition resistance in colon cancer with *KRAS* and *PIK3CA* mutations

**DOI:** 10.1038/s41416-019-0434-5

**Published:** 2019-04-04

**Authors:** Jai-Hee Moon, Seung-Woo Hong, Jeong Eun Kim, Jae-Sik Shin, Jin-Sun Kim, Soo-A Jung, Seung Hee Ha, Seul Lee, Joseph Kim, Dae Hee Lee, Yoon Sun Park, Dong Min Kim, Sang-Soo Park, Jun Ki Hong, Do Yeon Kim, Eun Ho Kim, Joonyee Jung, Mi Jin Kim, Seung-Mi Kim, Dustin A. Deming, Kyunggon Kim, Tae Won Kim, Dong-Hoon Jin

**Affiliations:** 1 Asan Institute for Life Science, Asan Medical Center, Seoul, Republic of Korea; 2 Department of Medical Science, University of Ulsan College of Medicine, Asan Medical Center, Seoul, Republic of Korea; 3 Department of Convergence Medicine, University of Ulsan College of Medicine, Asan Medical Center, Seoul, Republic of Korea; 4 Department of Oncology, University of Ulsan College of Medicine, Asan Medical Center, Seoul, Republic of Korea; 5 Department of Internal Medicine, University of Ulsan College of Medicine, Asan Medical Center, Seoul, Republic of Korea; 6 Division of Hematology and Oncology, Department of Medicine, University of Wisconsin-Madison, Madison, WI USA; 7 Department of Convergence Medicine, Convergence Medicine Research Center/Biomedical Research Center, Asan Medical Center, University of Ulsan College of Medicine, Seoul, Republic of Korea; 8 Department of Medical Sicence, Asan Medical Institute of Convergence Science and Technology, Asan Medical Center, University of Ulsan College of Medicine, Seoul, Republic of Korea

**Keywords:** Cancer therapeutic resistance, Tumour biomarkers, Colon cancer

## Abstract

**Background:**

Mitogen-activated protein kinases (MEK 1/2) are central components of the RAS signalling pathway and are attractive targets for cancer therapy. These agents continue to be investigated in *KRAS* mutant colon cancer but are met with significant resistance. Clinical investigations have demonstrated that these strategies are not well tolerated by patients.

**Methods:**

We investigated a biomarker of response for MEK inhibition in *KRAS* mutant colon cancers by LC-MS/MS analysis. We tested the MEK inhibitor in *PIK3CA* wild(wt) and mutant(mt) colon cancer cells. In addition, we tested the combinational effects of MEK and TNKS inhibitor in vitro and in vivo.

**Results:**

We identified β-catenin, a key mediator of the WNT pathway, in response to MEK inhibitor. MEK inhibition led to a decrease in β-catenin in *PIK3CA* wt colon cancer cells but not in mt. Tumour regression was promoted by combination of MEK inhibition and NVP-TNS656, which targets the WNT pathway. Furthermore, inhibition of MEK promoted tumour regression in colon cancer patient-derived xenograft models expressing *PIK3CA* wt.

**Conclusions:**

We propose that inhibition of the WNT pathway, particularly β-catenin, may bypass resistance to MEK inhibition in human *PIK3CA* mt colon cancer. Therefore, we suggest that β-catenin is a potential predictive marker of MEK inhibitor resistance.

## Background

Colon cancer has a high incidence, comprising ~13% of all cancers worldwide. Standard therapeutic approaches for treating colon cancer patients include combination regimens such as irinotecan**/**5-FU/leucovorin (FOLFIRI) or oxaliplatin/5-FU/leucovorin (mFOLFOX6).^1^ Treatment with molecularly targeted agents, such as bevacizumab and cetuximab, has improved overall survival in colon cancer patients; unfortunately, not all patients benefit from these targeted agents. Colon cancer patients with *KRAS* mutations do not respond to cetuximab or panitumumab, which are antibodies that target epidermal growth factor receptor (EGFR).^2–5^ Because these mutations are found in 40% of colon cancers,^6^ additional treatment options and biomarkers of response are urgently needed for *KRAS* mutant cancers.

Mitogen-activated protein kinase (MEK) is an essential component within the RAF/MEK/ERK pathway downstream of *KRAS*. MEK inhibition has the potential to block inappropriate signal transduction regardless of the upstream position of the oncogenic aberration;^6,7^ in addition, MEK inhibition suppresses cell growth and induced apoptosis. In *KRAS* mutant cancers, the phosphatidylinositol 3-kinase (PI3K) genotype influences the patient’s sensitivity to MEK inhibitors.^8^ *PIK3CA* mutations in various cancer cells correlate with resistance to MEK inhibitors, and cells transduced with PI3K mutant are resistant to MEK inhibition. *KRAS* stimulates multiple signalling effectors, including the PI3K pathway. Extensive crosstalk has been observed between the PI3K and RAS/RAF/MEK/ERK signalling pathways. Several studies have shown that the majority of MEK inhibitor-insensitive colon cancer cell lines harbour activating mutations in the PI3K pathway, whereas *KRAS* mutant cancer cells with an intact wild-type PI3K pathway are sensitive to MEK inhibitors.^9^

Recently, phase I clinical trials examined therapeutic approaches for the treatment of metastatic solid tumours using a combination of MEK inhibitors with PI3K/mTOR inhibitors.^10^ Most phase I clinical trials exploring these combinations have been unable to increase the doses of either agent to the respective individual maximal tolerated dose.

The WNT/β-catenin pathway is associated with embryonic development and cancer progression, and its activation is highly prevalent in colon cancer.^3^ A key feature of the Wingless-INT (WNT) pathway is the regulated proteolysis of the downstream effector β-catenin by the β-catenin destruction complex. Constitutive β-catenin signalling due to either inactivating mutations in APC or activating mutations within β-catenin itself also plays a critical role in the development of colon cancer; nearly 90% of all colon cancers harbour mutations that drive β-catenin signalling.^11^ Several small molecules that target the WNT pathway have been developed, and their inhibitory effects on tumour growth have been reported.^11,12^ Tankyrase inhibitors (TNKSi) induce stabilisation of AXIN, which abrogates WNT/β-catenin signalling and induces apoptosis. Although many groups have studied WNT/β-catenin-targeted therapies, many important problems remain unsolved regarding inhibition of this pathway.

In our study, we attempted to identify a biomarker of MEK inhibition in colon cancer cells. First, we confirmed that the PI3K genotype is a key factor in determining sensitivity to MEK inhibitors. Second, we identified and evaluated β-catenin as a biomarker. We demonstrated that β-catenin plays a major role in the cell response to MEK inhibition. Moreover, combinational treatment with TNKSi and MEK inhibitors led to apoptosis in MEK inhibitor-resistant cells. Taken together, our results suggest that β-catenin is a novel predictive pharmacodynamic (PD) biomarker of MEK inhibitor resistance and a potential target for combinatorial treatment regimens.

## Materials and methods

### Cell culture

Human colon cancer cells were purchased from ATCC (Manassas, VA, USA) or the Korea Cell Bank (KCLB, Seoul, Republic of Korea). The cells were cultured in RPMI medium or DMEM (WelGene Co., Daegu, Republic of Korea) supplemented with 10% fetal bovine serum (FBS) and penicillin/streptomycin (100 μg/ml) (Invitrogen, Carlsbad, USA) and maintained at 37 °C in an atmosphere containing 5% CO_2_. The DLD-1 isogenic cell lines *PIK3CA* wild-type (351) and *PIK3CA* mutant (353) were provided by Dr. Vogelstein, cultured in McCoy’s medium (WelGene Co., Daegu, Republic of Korea) supplemented with 10% FBS and penicillin/streptomycin (100 μg/ml), and maintained at 37 °C in an atmosphere containing 5% CO_2_. Additionally, the HCT116 isogenic cell lines β-catenin parent (wild-type/mutant, 54), β-catenin mt (mutant/-, with wt allele knockout, 240), and β-catenin wt (wild-type/-, with mutant allele knockout, 241) were provided by Dr. Vogelstein and cultured in McCoy’s medium supplemented with 10% FBS and penicillin/streptomycin.

### Cell death

Cells were seeded and treated with the indicated dose of MEK inhibitor (AZD6244, GSK112012, AS703026, or BAY 86-9766) (Selleckchem, Houston, TX). After 24 h, the cells were harvested and evaluated with the Trypan blue exclusion assay. Additionally, DLD-1, HCT-8, DLD-1 isogenic cells (*PI3K* mutant: 353), and HCT116 isogenic cells (54, 240, and 241) were plated and treated with 5 μM NVP-TNKS656 (tankyrase inhibitor) and 1 µM GSK112012 (MEK inhibitor). After 48 h, the cell death rate was determined with the Trypan blue exclusion assay.

### Plasmids, siRNA, and transfection

Plasmids containing wild-type or mutant β-catenin (pCI-neo-β-catenin wt and pCI-neo-β-catenin mutant delta45, Addgene, Cat# 16518 and 16520; pcDNA 3.1 β-catenin wt) or siRNA/shRNA were transfected with Lipofectamine 2000 or Lipofectamine RNAiMAX (Invitrogen, Carlsbad, CA, USA), as appropriate according to the manufacturer’s instructions. The sequence used in the siRNA/shRNA transfection studies is AACTATCAAGATGATGCAGAACTTGCC. shRNAs against β-catenin were acquired from Genolution (Seoul, Republic of Korea).

### Colony-forming assay

Clonogenic capacity was evaluated by plating vehicle- or MEK inhibitor-treated cells at a density of 100 cells per six-well plate. At 10–14 days after treatment, the cells were fixed and stained with 0.01% crystal violet solution.

### Western blot analysis

Cells were harvested and lysed in RIPA buffer (Cell Signalling Technology, Danvers, MA), and the resultant proteins were separated by sodium dodecyl sulphate-polyacrylamide gel electrophoresis (SDS-PAGE) and transferred to PolyScreen membranes (NEN, Boston, MA, USA). The membranes were blocked with 5% non-fat dry milk in Tris-buffered saline containing 0.05% Tween-20 (TBS-T) and probed with antibodies targeting the following proteins: β-catenin (Cell Signalling Technology), cyclin D1 (Santa Cruz Biotechnology, Santa Cruz, CA, USA), AXIN2, c-Myc, STAT3, cyclin B1, Gab1, c-caspase-3, p-ERK (Cell Signalling Technology), HA, β-actin, and γ-tubulin (Santa Cruz Biotechnology). Primary antibodies were detected with HRP-conjugated anti-mouse or anti-rabbit antibodies, as appropriate, that were subjected to enhanced chemiluminescence (Amersham, Buckinghamshire, UK).

### Annexin V/propidium iodide (PI) staining

Cells were harvested, washed twice in PBS, and resuspended in 100 µl of annexin binding buffer (BD Pharmingen). Then, these cells were stained with 5 µl of annexin V and 1 µg/ml PI. After staining, 300 µl of annexin-binding buffer was added, and the stained cells were analysed using a flow cytometer (BD Bioscience).

### Protein identification by LC–MS/MS

SW620 and DLD-1 cells cultured in the presence or absence of MEK inhibitor were harvested and lysed. After the protein concentration was detected with the BCA assay, SDS-PAGE, in-gel digestion, acidic/basic extraction, and DK-tip desalting for drug-treated cells were performed. Finally, the proteins were analysed with Triple TOP DDA or SWATH.

### In vivo tumour xenografts and immunohistochemical analysis

Xenograft tumour growth in BALB/c nude mice was used as a model for in vivo experiments. All animal studies were conducted in compliance with animal protocols approved by the Asan Medical Centre Institutional Animal Care and Use Committee. SW620, DLD-1, and DLD-1 isogenic cells (351 and 353) were subcutaneously implanted. When tumour sizes reached 100 mm^3^, the mice were administered the indicated doses of MEK inhibitor (oral administration; P.O. with or without NVP-TNKS656 (i.p.) for 28 days, and then the tumour volumes were monitored for 4 weeks (measured every 3 days). The tumour size was calculated as length × width^2^ × 0.5. After 28 days, the tumours were harvested, visualised, and measured. In addition, the tumours were analysed by western blot and immunohistochemistry, as described previously.^13^ In the patient-derived xenograft (PDX) model, tumour tissues were subcutaneously engrafted on the flank of 5-week-old female BALB/c nude mice. When tumour sizes reached 100 mm^3^, the mice were administered the indicated doses of MEK inhibitor (oral administration; P.O.) for 28 days. The tumour size and body weights were measured every 3 days for 4 weeks.

### Statistical analysis

All resulting data were statistically analysed using a two-tailed Student’s *t*-test, and the level of significance stated in the text was based on *P* values; a *P* value lower than 0.05 was considered statistically significant.

## Results

### β-Catenin is a potent biomarker candidate for alleviating MEK inhibitor resistance in *KRAS* mutant colon cancer cells with *PIK3CA* mutations

**Fig. 1:**
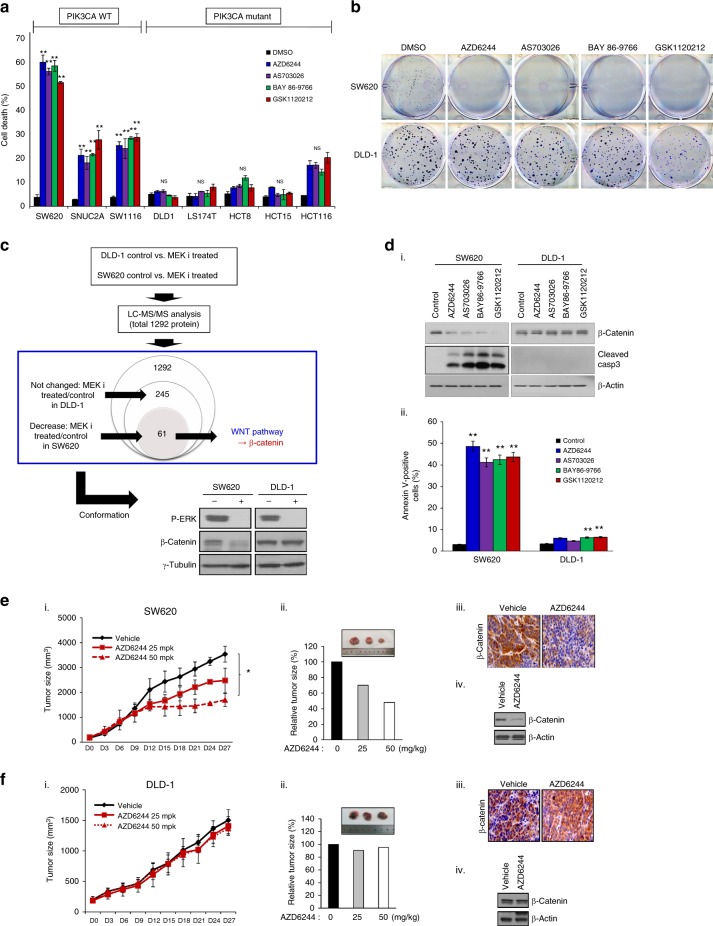
The *PIK3CA* genotype is critical for the response of colon cancer cells to MEK inhibitor treatment. **a** Colon cancer cells were treated with 1 μM MEK inhibitor (as indicated) for 24 h, and cell death was determined with the trypan blue exclusion assay. The data represent the means ± SDs of at least three independent experiments. All resulting data were statistically analysed using a two-tailed Student’s *t*-test *P < 0.05, **P < 0.01. **b** SW620 and DLD-1 cells were treated with 1 μM MEK inhibitor for 24 h and then reseeded at 1 × 10^2^ cells/well. After 2 weeks, the cells were washed, fixed, and stained for colony-forming assay. **c** Top panel: Overall results of LC-MS/MS analysis are shown as the identification process of β-catenin in determining sensitivity to MEK inhibitors. Lower panel: SW620 and DLD-1 cells were treated with 1 μM AZD6244 for 24 h, and the cell lysates were prepared for western blot analysis using the indicated antibodies. γ-Tubulin was used as a loading control. **d** SW620 and DLD-1 cells were treated with the indicated MEK inhibitors for 24 h. (i) β-Catenin expression was determined by western blot analysis using an antibody targeting β-catenin. In addition, we investigated cleaved caspase-3 expression in MEK inhibitor-treated SW620 and DLD-1 cells. β-Actin was used as a loading control. (ii) MEK inhibitor-treated cells were washed with PBS, stained with annexin V staining solution and analysed by flow cytometry. All resulting data were statistically analysed using a two-tailed Student’s *t*-test. *P < 0.05*,* **P < 0.01. **e** SW620 and **f** DLD-1 cells were subcutaneously injected into nude mice. Once the tumours grew to 100 mm^3^, the mice were treated with 25 or 50 mg/kg AZD6244, an MEK inhibitor, for 27 days. Each group comprised 4–5 mice. (i) Tumour growth was evaluated every 3 days. The data represent the mean ± SD. (ii) Relative tumour size was evaluated. (iii and iv) Representative photos are shown (inset figures). All resulting data were statistically analysed using a two-tailed Student’s *t*-test. *P < 0.05, **P < 0.01. After administration, we harvested tumour tissues, which were then lysed or fixed. We analysed β-catenin expression by immunohistochemistry or western blot analysis in tumour samples from AZD6244-treated (50 mg/kg) or vehicle groups. β-Actin was used as a loading control

To determine the sensitivity of the different cell lines to MEK inhibitors, we first exposed a panel of eight human colon cancer cell lines with *KRAS* mutations, i.e., three *PIK3CA* wild-type cell lines and five mutant cell lines, to a variety of selective MEK inhibitors, i.e., AZD6244, AS703026, BAY 86–9766, and GSK112012.^6,14^ These cells were treated with these four MEK inhibitors at the indicated concentrations. Wild-type *PIK3CA* colon cancer cells displayed sensitivity to each of the MEK inhibitors; however, the mutant *PIK3CA* colon cancer cells did not respond to the MEK inhibitors (Fig. 1a and Supplementary Table S1). Consistently, colony formation was dramatically decreased in wild-type *PIK3CA* cells following treatment with MEK inhibitors but did not decrease in cells that express mutant *PIK3CA* (Fig. 1b). Based on these findings, we aimed to identify proteins associated with this resistance to MEK inhibition in *KRAS* and *PIK3CA* mutant cancers. To accomplish this, we used LC–MS/MS. In DLD-1 cells cultured in the presence or absence of an MEK inhibitor, no significant changes in protein expression were identified for the 245 proteins analysed. In the SW620 cell line, 61 proteins were differentially expressed upon MEK inhibition. We have found the genes involved in cell proliferation or cellular signalling. Also, we investigated whether these proteins were related to the MEK pathway. But, most genes hardly have any reported information. In the case of β-catenin among 61 cases, there was a recent report confirming the relationship between the MEK pathway and the WNT pathway. Kang and co-workers^15^ demonstrated that β-catenin plays an important role in tumorigenesis (Supplementary Table S2). Next, we confirmed these results by western blot analysis. β-Catenin expression was decreased in MEK inhibitor-treated SW620 cells but unchanged in MEK inhibitor-treated DLD-1 cells (Fig. 1c). However, we demonstrated with RT-PCR that the mRNA expression levels of β-catenin were not changed after treatment with MEK inhibitor in *PIK3CA* wild-type cell lines (Supplementary Figure S1). To validate this finding, we selected two colon cancer cell lines with different *PIK3CA* genotypes, SW620, and DLD-1. We first examined the inhibitory effect of selective MEK inhibitors on β-catenin expression and observed that β-catenin expression in SW620 cells, which express wild-type *PIK3CA*, was clearly decreased in response to MEK inhibitor. In contrast, β-catenin expression was unchanged in DLD-1 cells, which express mutant *PIK3CA*, following treatment with MEK inhibition. Cytoplasmic staining for β-catenin was performed in SW620 and DLD-1 cell lines in the presence or absence of MEK inhibitor treatment. After MEK inhibitor treatment of SW620 cells, β-catenin expression was decreased; however, in the DLD-1 cells, β-catenin expression was unchanged (Supplementary Figure S2). Next, we investigated WNT signalling activity using a TOP/FOP flash assay. The results showed that relative luciferase activity (TOP flash activity) was decreased by MEK inhibition in SW620 cells. However, the relative luciferase activity was not changed by MEK inhibitor treatment of DLD-1 cells (Supplementary Figure S3). In addition, caspase-3 cleavage was observed in colon cancer cells that express wild-type *PIK3CA* (Fig. 1d, i). This result was concomitant with annexin V-positive cell populations (Fig. 1d, ii). We investigated the proportion of cells in late apoptosis and early apoptosis following treatment with MEK inhibition or control. For SW620 cells late and early apoptosis were similar between the treatment groups (Supplementary Figure S4). Consistent with this observation, tumour growth in the *PIK3CA* wild-type SW620-bearing xenograft model was gradually reduced response to treatment with the selective MEK inhibitor, AZD6244, whereas tumour growth was not slowed in the *PIK3CA* mutant DLD-1-bearing xenograft model (Fig. 1e, f). In conjunction with these results, β-catenin expression was decreased in tissues from SW620-derived tumours based on immunohistochemical and western blot analyses, whereas β-catenin expression in tissues from DLD-1-derived tumours was not altered. Thus, differences in sensitivity to MEK inhibitors may depend on the *PIK3CA* genotype in human colon cancer cells.

### Sensitivity to MEK inhibition depends on β-catenin expression

**Fig. 2:**
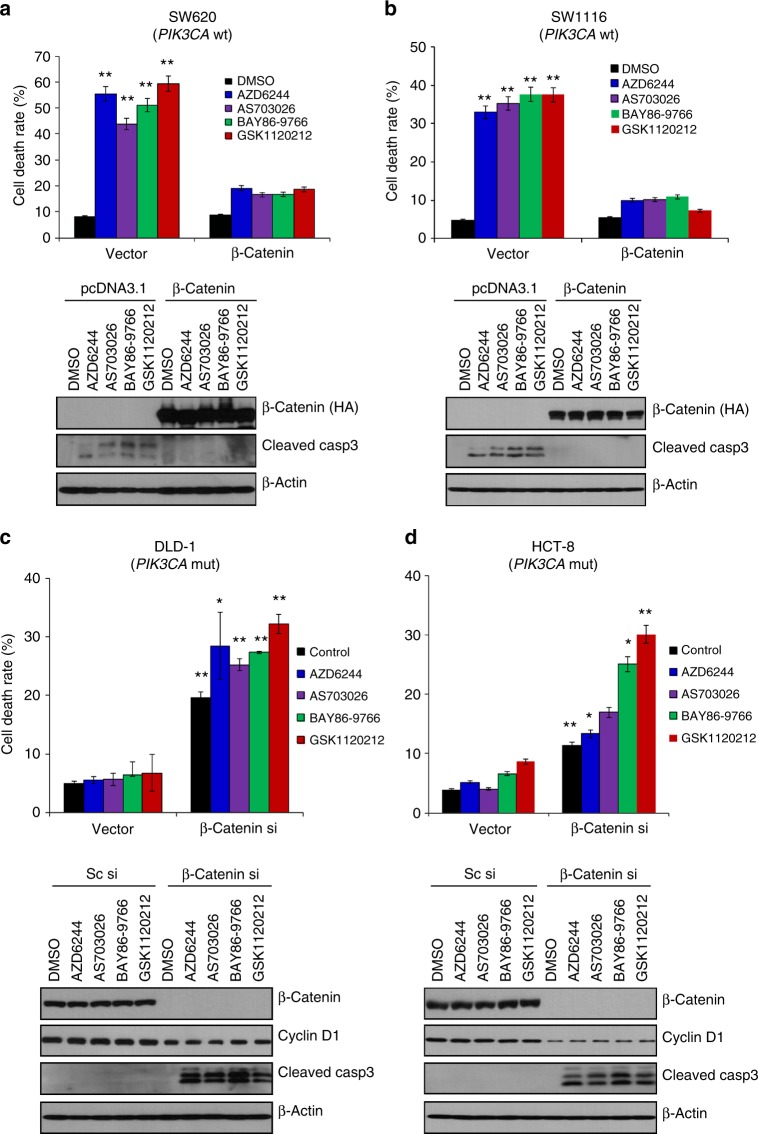
β-Catenin expression determines MEK inhibitor sensitivity. SW620 (**a**) and SW1116 cells (**b**) expressing wild-type *PIK3CA* were transfected with pcDNA3.1 plasmid containing empty vector or β-catenin for 24 h and then treated with various MEK inhibitors (1 µM) for another 24 h. **a**, **b** (Upper panel) Cell death was determined with the Trypan blue exclusion assay. The data represent the means ± SDs of at least three independent experiments. **a**, **b** (Lower panel) All resulting data were statistically analysed using a two-tailed Student’s *t*-test *P *<* 0.05*,* **P *<* 0.01*.* Cell lysates were used for western blot analysis using antibody targeting the HA tag or cleaved caspase-3. β-Actin was used as a loading control. DLD-1 (**c**) and HCT-8 *PIK3CA* mt cells (**d**) were transfected with scramble or β-catenin siRNA for 24 h and then treated with the indicated MEK inhibitors for another 24 h. **c**, **d** (Upper panel) Cell death was evaluated with the Trypan blue exclusion assay. The data represent the means ± SDs of at least three independent experiments. **c**, **d** (Lower panel). Western blot analysis was performed using antibodies targeting β-catenin, cyclin D1, cleaved caspase-3, and β-actin, which was used as a loading control. All resulting data were statistically analysed using a two-tailed Student’s *t*-test. *P < 0.05, **P < 0.01

To further determine whether the difference in colon cancer cell sensitivity to MEK inhibitors is a result of decreased β-catenin expression, we first examined the effect of β-catenin overexpression on MEK inhibitor-treated colon cancer cells that express wild-type *PIK3CA*. Briefly, a pcDNA3.1 plasmid containing wild-type β-catenin cDNA with an HA tag was constructed. Initially, SW620 cells expressing wild-type *PIK3CA* were transfected with a construct containing wild-type β-catenin cDNA and then subjected to MEK inhibitor treatment. Improved cell viability was observed in cells transfected with β-catenin compared to that in control vector-transfected cells (Fig. 2a). In addition, decreased cell death was observed in SW1116 colon cancer cells, which express wild-type *PIK3CA*, transfected with β-catenin cDNA (Fig. 2b). Consistent with these data, cleaved caspase-3 was not detected in β-catenin cDNA-transfected cells. Thus, the differential sensitivities to MEK inhibitors may result from decreased β-catenin expression.

To ascertain the dependence of MEK inhibitors on β-catenin, we investigated the effect of β-catenin silencing via small interfering RNA (siRNA) on MEK inhibitor-treated colon cancer cells that express mutant *PIK3CA*. We used a single siRNA sequence for β-catenin knockdown, which was analysed by a BLAST programme and confirmed for its specificity to β-catenin. We confirmed the inhibition of β-catenin expression at the mRNA and protein levels (Supplementary Figure S5, Fig. 2c, d). β-Catenin knockdown led to increased levels of cell death in the colon cancer cell line DLD-1, which expresses mutant *PIK3CA*, in the presence of an MEK inhibitor (Fig. 2c). Additionally, cell death increased significantly after MEK inhibitor exposure in β-catenin siRNA-treated HCT-8 cells, which are *PIK3CA* mutant colon cancer cell lines (Fig. 2d). In addition, cleaved caspase-3 was detected in these cells but not in the scrambled siRNA-treated cells. Furthermore, we investigated the combinational effects of the tankyrase inhibitor NVP-TNS656 and the MEK inhibitor GSK112012 on HCT116 isogenic cell lines (54, 240, and 241). In the case of cell line 241 (the β-catenin wild-type), increased death rates were observed following cotreatment with NVP-TNKS656 and GSK112012. However, the β-catenin mutant (#240) and β-catenin wt/mt (#54) cell lines did not respond to cotreatment with NVP-TNKS656 and GSK112012. These results indicate that the *PIK3CA* genotype-dependent differential sensitivity to MEK inhibitors is also dependent on the β-catenin genotype.

### Pharmacological inhibition of β-catenin can overcome MEK inhibitor resistance in *KRAS* and *PIK3CA* mutant colon cancer cells

**Fig. 3:**
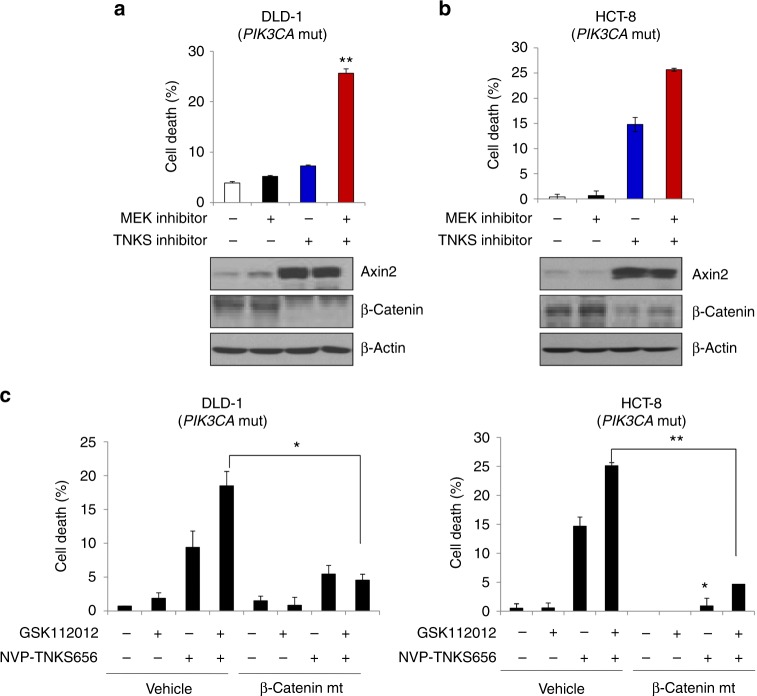
Pharmacological inhibition of β-catenin overcomes MEK inhibitor resistance in *PIK3CA* mutant colon cancer cells. DLD-1 (**a**) and HCT-8 cells (**b**) were treated with 1 µM AZD6244 and/or 5 µM NVP-TNKS656 for 24 h. **a**, **b** (Upper panel) The combined effect of AZD6244 and NVP-TNKS656 is shown by the Trypan blue exclusion assay. The data represent the means ± SDs of at least three independent experiments. **a**, **b** (Lower panel) Expression levels of β-catenin and Axin2 were verified by western blot analysis. All resulting data were statistically analysed using a two-tailed Student’s *t*-test. *P < 0.05, **P < 0.01. **c** (Left panel) DLD-1 and (right panel) HCT-8 cells were transfected with empty vector or constitutively active β-catenin for 24 h and then treated with 1 µM GSK112012, an MEK inhibitor, and/or 5 µM NVP-TNKS656, a tankyrase inhibitor, for another 24 h. Cell death was determined with the Trypan blue exclusion assay. The data represent the means ± SDs of at least three independent experiments. All resulting data were statistically analysed using a two-tailed Student’s *t*-test. *P < 0.05, **P < 0.01

Recently, NVP-TNKS656 and XAV939 were reported to negatively regulate the protein stability of β-catenin through inhibition of tankyrase 1/2, which interacts with a highly conserved domain of AXIN and stimulates its degradation through the ubiquitin–proteasome pathway.^2^ Therefore, we investigated the combinatorial effect of a TNKS inhibitor NVP-TNKS656 with MEK inhibition in *KRAS* and *PIK3CA* mutant colon cancer cells. DLD-1 and HCT-8 cells, two colon cancer cell lines with mutant *PIK3CA* expression, were selected. Treatment with NVP-TNKS656 and the MEK inhibitors AZD6244 or GSK112012 significantly induced increases in cell death in both cell lines (Fig. 3a, b). In contrast, monotherapy with an MEK inhibitor (AZD6244 or GSK112012) or NVP-TNKS656 alone did not significantly alter cellular viability. To further examine the dependence of the inhibitory effects of NVP-TNKS656 on β-catenin, we constructed a plasmid expressing a β-catenin mutant (Ser33 replaced with Tyr) that does not induce proteasome-dependent degradation.^16^ Compared with mutant *PIK3CA* cells transfected with control vector, the corresponding cells transfected with this β-catenin mutant resulted in decreased cell death after cotreatment with the MEK inhibitor GSK112012 and NVP-TNKS656 (Fig. 3c). These results suggest that pharmacological inhibition of β-catenin together with MEK inhibition may be a potent combinatorial therapeutic strategy for treating colon cancer cells that express both mutant *PIK3CA* and mutant *KRAS*.

**Fig. 4:**
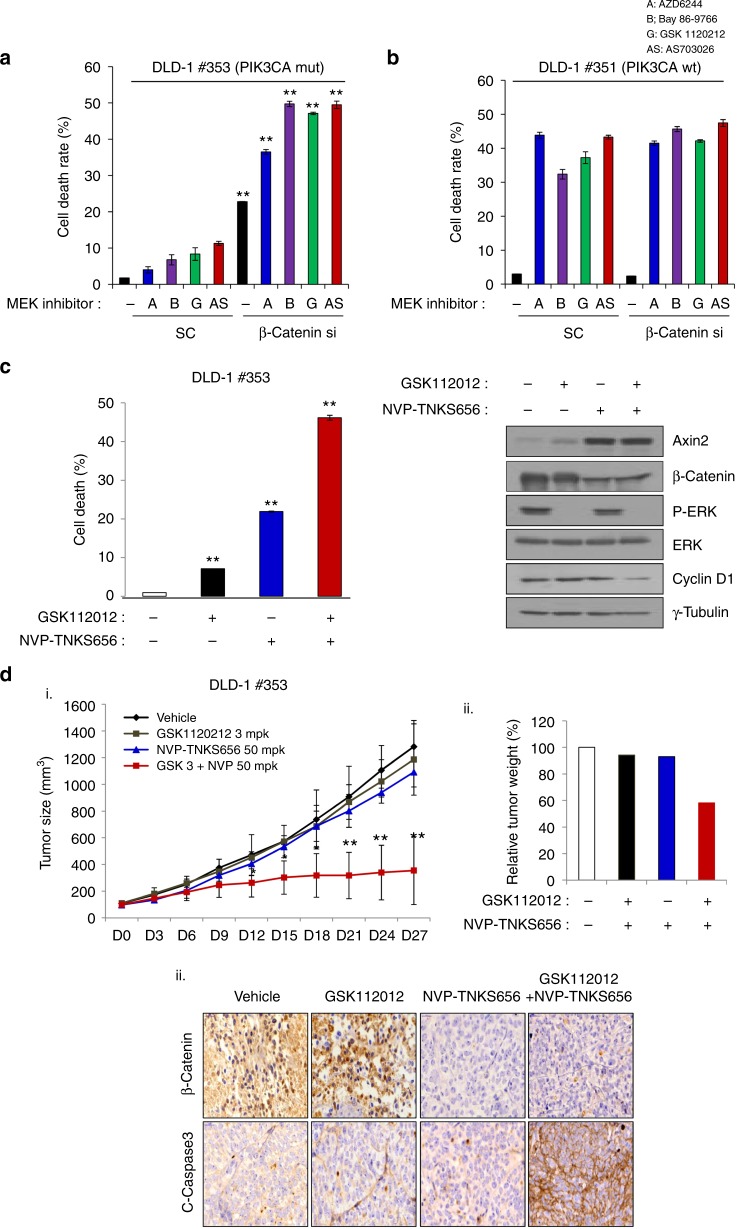
Inhibition of β-catenin in DLD-1–*PIK3CA* isogenic cells is determined by their sensitivity to MEK inhibitors. DLD-1–*PIK3CA* mt (cell line 353) (**a**) and DLD-1–*PIK3CA* wt (cell line 351) isogenic cells (**b**) were transfected with scramble siRNA or β-catenin siRNA for 48 h and then treated with the indicated 1 µM MEK inhibitors for another 24 h. MEK inhibitor resistance was overcome by β-catenin knockdown in *PIK3CA* mutant cells as shown by the Trypan blue exclusion assay. The data represent the means ± SDs of at least three independent experiments. All resulting data were statistically analysed using a two-tailed Student’s *t*-test. *P < 0.05, **P < 0.01 **c** Combinatorial effect of the MEK inhibitor GSK112012 and the β-catenin pharmacological inhibitor NVP-TNKS656 on DLD-1-*PIK3CA* mt (cell line 353) cells were evaluated with the Trypan blue exclusion assay. Cell lysates were used to analyse Axin2, β-catenin, p-ERK, ERK, and cyclin D1 expression by western blot analysis. γ-Tubulin was used as a loading control. All resulting data were statistically analysed using a two-tailed Student’s *t*-test. *P < 0.05, **P < 0.01. **d** Tumour from a nude mouse subcutaneously injected with DLD-1-*PIK3CA* mt (cell line 353) cells. (i) When the tumour volume reached 100 mm^3^, the tumours were treated daily with 3 mg/kg GSK112012, an MEK inhibitor, and/or 50 mg/kg NVP-TNKS656, a tankyrase inhibitor. Tumour size was measured every 3 days after drug treatment. The data represent the means ± SDs. Each group comprised 4–5 mice. All resulting data were statistically analysed using a two-tailed Student’s *t*-test *P < 0.05, **P < 0.01. (ii) Tumour weight was assessed after 27 days of drug treatment. (iii) β-Catenin and cleaved caspase-3 expression was confirmed by immunohistochemical analysis

To ascertain the effect of MEK inhibition on β-catenin status in *PIK3CA* mutant colon cancer cells, we used two special colon cancer cell lines: DLD-1–*PIK3CA* wt (cell line 351), which has the mutant allele knocked out, and DLD-1–*PIK3CA* mt (cell line 353), which has the wt allele knocked out. MEK inhibitor treatment of cell line 353 after transfection with scrambled shRNA did not induce cell death. In contrast, cell line 353 with β-catenin knockdown displayed increased cell death in response to MEK inhibitor (Fig. 4a). These results coincided with the data shown in Fig. 2c; however, all MEK inhibitors significantly induced cell death in cell line 351 after transfection with scrambled shRNA (Fig. 4b). Notably, no combinatorial effects were observed in DLD-1–*PIK3CA* wt cells with β-catenin knockdown and MEK inhibitor treatment. To further examine the combinatorial effects of β-catenin inhibition and MEK inhibitor treatment, we treated cell line 353 with both the MEK inhibitor GSK112012 and the WNT pathway inhibitor NVP-TNKS656. Treatment of cell line 353 with NVP-TNKS656 and the GSK112012 led to significantly increased cell death compared with that in cells treated with either drug alone (Fig. 4c). In addition, we investigated the expression of β-catenin, AXIN2, p-ERK, ERK, and cyclin D1 by western blot analysis. These results showed that the expression levels of β-catenin and cyclin D1 were decreased after cotreatment with NVP-TNKS656 and MEK inhibitor. Furthermore, we examined WNT signalling activity with the TOP/FOP flash assay. These results showed that TOP flash activity decreased secondary to the combinational treatment (with NVP-TNKS656 and MEK inhibitor) in DLD-1 isogenic cell lines (Supplementary Figure S6). Therefore, these results indicate that *PIK3CA* mt, but not wt, cancers are dependent on β-catenin signalling to overcome MEK inhibitor treatment and this is a new target for therapeutic strategies.

Based on the above data, we next examined the effect of oral administration of NVP-TNKS656 and the MEK inhibitor GSK112012 on the growth of tumours derived from cell line 353 in nude mice. Each inhibitor treatment was initiated after the tumours reached ~100 mm^3^ in volume. The growth of cell line 353 tumours that express mutant *PIK3CA* was significantly suppressed by cotreatment with both inhibitors, whereas no effect of monotherapy on cell line 353 tumours was detected (Fig. 4d). Consistently, increased levels of cleaved caspase-3 and decreased levels of β-catenin were only observed in tissues treated with both inhibitors. These results suggest that pharmacological inhibition of β-catenin can bypass MEK inhibitor resistance in *PIK3CA* mutant colon cancer cells and that this approach may be a potent combination therapeutic strategy.

### *PIK3CA* genotype-dependent anti-tumour effects of MEK inhibition on a colon cancer PDX model

**Fig. 5:**
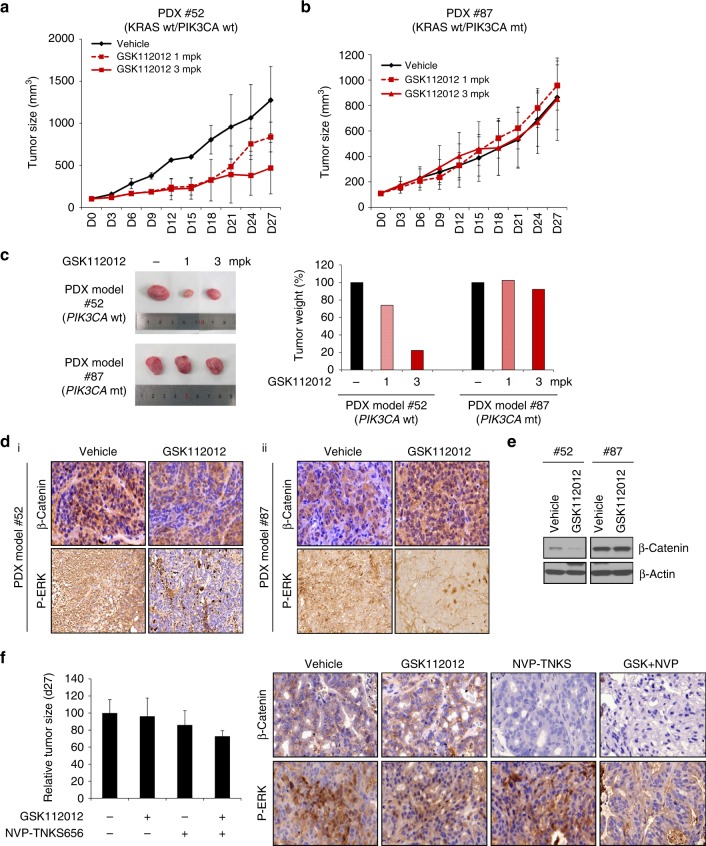
β-Catenin is a determinant factor of MEK inhibitor sensitivity in PDX models. **a**, **b** Tumours were grown in PDX 52 (*PIK3CA* wt) and PDX 87 (*PIK3CA* mt) models. When the tumour volume reached 100 mm^3^, the mice were treated daily with GSK112012, an MEK inhibitor. Tumour size for **a** PDX 52 (*PIK3CA* wt) and **b** PDX 87 (*PIK3CA* mt) was measured every 3 days after treatment. Each group comprised 4–5 mice. The data represent the means ± SDs. All resulting data were statistically analysed using a two-tailed Student’s *t*-test. *P < 0.05, **P < 0.01. **c** (Left panel) At 27 days after MEK inhibitor treatment, the tumours were imaged. (Right panel) The effect of MEK inhibitor on tumour weight in PDX models was evaluated. **d** Expression of β-catenin and p-ERK in GSK112012-treated PDX models by immunohistochemical analysis. **e** Expression of β-catenin and p-ERK in GSK112012-treated PDX models was analysed by western blot using β-catenin and p-ERK antibodies, respectively. β-Actin was used as a loading control. **f** At 27 days after initiation of MEK inhibitor and NVP-TNKS656 cotreatment, the tumour size was reduced (left panel). The right panel shows that β-catenin and p-ERK protein expression was decreased in the group cotreated with MEK inhibitor and NVP-TNKS656

As the above data show, decreased levels of β-catenin were observed only in human colon cancer cells expressing wild-type *PIK3CA*, suggesting that β-catenin may be a predictive PD marker of MEK inhibitor resistance. To confirm this possibility, we used human colon cancer PDX models with cell line 52, which expresses wild-type *KRAS* and *PIK3CA*, and cell line 87, which expresses wild-type *KRAS* and mutant *PIK3CA*. When the tumours reached ~100 mm^3^ in volume, MEK inhibitor (GSK112012) was orally administered to mice carrying these PDXs. Tumour growth and tumour weight decreased in the 52 PDX model following GSK112012 treatment (Fig. 5a, c) but were not affected in the 87 PDX model (Fig. 5b, c). Furthermore, we confirmed β-catenin expression in the PDX tissues. Immunohistochemical and western blot analyses showed that β-catenin was significantly decreased in 52 PDX tissues at 28 days after GSK112012 treatment, but that 87 PDX tissues were not affected by this treatment (Fig. 5d, e). Consistent with these data, decreased phospho-ERK levels were also observed. Furthermore, tumour growth decreased in the mutant *PIK3CA–*PDX model following administration of GSK112012 (3 mg/kg) and NVP-TNKS656 (50 mg/kg), the latter of which targets the WNT pathway (Fig. 5f). After administration, we harvested tumour tissues and analysed β-catenin and p-ERK expression by western blot. These results showed that β-catenin expression was decreased after treatment with NVP-TNKS656 and GSK112012 (Fig. 5f, right panel). In addition, we tested the effects of GSK112012 (3 mg/kg) and NVP-TNKS656 (100 mg/kg) in the PDX model (#87). Tumour growth was dramatically inhibited by GSK112012 and NVP-TNKS656. Unfortunately, mice in these groups almost died after 6 days due to a 20% loss of body weight. In the cases of single drug treatment (GSK112012 or NVP-TNKS656), tumour size was not changed (Supplementary Figure S8). These results suggest that the difference in MEK inhibitor sensitivity is dependent on the *PIK3CA* genotype and that β-catenin is a potential PD marker for MEK inhibitor resistance. Additionally, these results demonstrate that in vivo therapeutic benefit for the combination of MEK and TNKS inhibition in *KRAS* and *PIK3CA* mt cancers.

## Discussion

MEK inhibitors are actively being developed for cancers with constitutive activation of the RAS/RAF/MEK pathway.^6^ RAS mutant cancers exhibit a variable response to these compounds, with intrinsic resistance commonly encountered in clinical trials.^17^ Many MEK inhibitor-resistant cell lines harbour activating mutations in the PI3K pathway, including *PIK3CA*.^9^ This has resulted in investigations into dual inhibition of the MEK and PI3K pathways; however, these combinations have been limited in their clinical usefulness due to significant toxicities.

Constitutive β-catenin signalling due to inactivating mutations in *APC* or activating mutations within β-catenin itself plays a critical role in colon cancer development. Therefore, the β-catenin signalling pathway is another potential target for colon cancer treatment.^11^ Here, we demonstrate that MEK inhibitor sensitivity was driven by the *PIK3CA* genotype in *KRAS* mutant colon cancer cells. MEK inhibitor treatment induced cell death in *PIK3CA* wt cancer cells, whereas *PIK3CA* mt cells did not respond to MEK inhibitor treatment. Thus, we examined which factor determined MEK inhibitor sensitivity. We used LC-MS/MS analysis and determined that β-catenin plays an important role in determining MEK inhibitor sensitivity. These results were consistent with previous reports; namely, several studies have also identified WNT signalling as a resistance mechanism in MAPK pathway-activated colorectal cancer (CRC) cells. In addition, we demonstrated that β-catenin overexpression decreased cell death in MEK inhibitor-treated cells. Moreover, β-catenin expression was significantly decreased in the *PIK3CA* wild-type PDX model (#52) upon MEK inhibitor administration. In contrast, β-catenin expression did not change in the *PIK3CA* mutant PDX model upon MEK inhibitor administration. Thus, our results suggest that β-catenin is a predictive biomarker of MEK inhibitor sensitivity in colon cancer.

β-Catenin plays an important role in the WNT/β-catenin signalling pathway and is involved in both intracellular signalling and cell adhesion. Mutations of the β-catenin gene presumably disrupt these functions, leading to cell proliferation.^18–20^ In our study, we investigated possible approaches to overcoming MEK inhibitor resistance in colon cancer cells. We demonstrated β-catenin to be a key factor in the resistance of colon cancer cells to MEK inhibitors. β-Catenin was inhibited by the tankyrase inhibitor NVP-TNKS656, and combined treatment of NVP-TNKS656 and MEK inhibitor induced cell death in DLD-1 and HCT-8 (*PIK3CA* mt) cells. However, β-catenin mutant cells did not respond to the cotreatment of NVP-TNKS656 and MEK inhibitor. The combinatorial treatment also induced cell death in the DLD-1 isogenic *PIK3CA* mt cell line 353. In addition, we tested the combinatorial effects of GSK112012 and NVP-TNKS656 on HCT116-β-catenin isogenic cells, including 54 (parent as a control), 240 (β-catenin mt/mt) and 241 (β-catenin wt/-). NVP-TNKS656 alone and NVP-TNKS656/MEK inhibitor combination treatments induced cell death in 241 cells. However, neither 54 nor 240 responded to the same treatments (Supplementary Figure S7). Therefore, our results demonstrated that NVP-TNKS656 is able to overcome MEK inhibitor resistance in *KRAS* mutant and *PIK3CA* mt/β-catenin wt colon cancer cells.

**Fig. 6:**
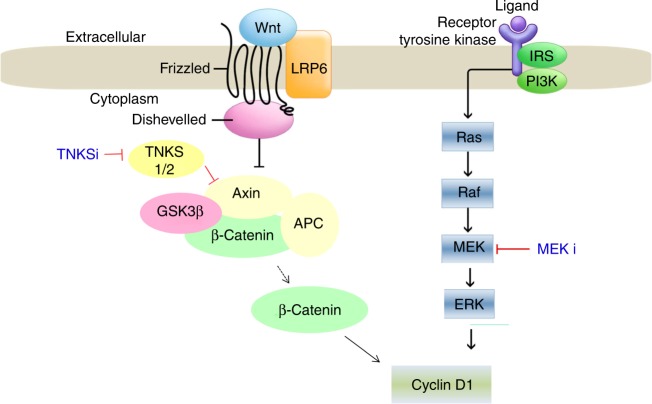
Summary for our hypothesis in MEK inhibitor-resistant *PIK3C*A mt colon cancer cells. These figures show that treatment with tankyrase inhibitors can help *PIK3CA* mutant colon cancer cells overcome MEK inhibitor resistance

In conclusion, our study identified β-catenin signalling as an important factor in MEK inhibitor resistance in *KRAS* mutant colon cancers. More importantly, combinatorial treatments such as NVP-TNKS656 and MEK inhibitor greatly induce cell death in a subset of *KRAS* mt/*PIK3CA* mt/β-catenin wt cells. Figure 6 displays a working model based on our results. MEK inhibitor-resistant cells were overcome by treatment with tankyrase inhibitor because cyclin D1 expression was decreased (Fig. 6). Therefore, our findings indicated that β-catenin is a potential biomarker of MEK inhibitor sensitivity in human colon cancer cells.

## Supplementary information

Supplementary Figure and Table legend

Supplementary Figure S1

Supplementary Figure S2

Supplementary Figure S3

Supplementary Figure S4

Supplementary Figure S5

Supplementary Figure S6

Supplementary Figure S7

Supplementary Figure S8

Supplementary Table S1

Supplementary Table S2

## Data Availability

Materials and data are available from the corresponding author upon reasonable request.

## References

[1] Kamnerdsupaphon P, Lorvidhaya V, Chitapanarux I, Tonusin A, Sukthomya V (2007). FOLFIRI chemotherapy for metastatic colorectal cancer patients. J. Med. Assoc. Thail..

[2] Aberle H, Bauer A, Stappert J, Kispert A, Kemler R (1997). beta-catenin is a target for the ubiquitin-proteasome pathway. EMBO J..

[3] Clevers H (2006). Wnt/beta-catenin signaling in development and disease. Cell.

[4] Pietrantonio F, Petrelli F, Coinu A, Di Bartolomeo M, Borgonovo K, Maggi C (2015). Predictive role of BRAF mutations in patients with advanced colorectal cancer receiving cetuximab and panitumumab: a meta-analysis. Eur. J. Cancer.

[5] Schoumacher M, Hurov KE, Lehar J, Yan-Neale Y, Mishina Y, Sonkin D (2014). Inhibiting Tankyrases sensitizes KRAS-mutant cancer cells to MEK inhibitors via FGFR2 feedback signaling. Cancer Res..

[6] Yoon J, Koo KH, Choi KY (2011). MEK1/2 inhibitors AS703026 and AZD6244 may be potential therapies for KRAS mutated colorectal cancer that is resistant to EGFR monoclonal antibody therapy. Cancer Res..

[7] Wang JY, Wilcoxen KM, Nomoto K, Wu S (2007). Recent advances of MEK inhibitors and their clinical progress. Curr. Top. Med. Chem..

[8] Halilovic E, She QB, Ye Q, Pagliarini R, Sellers WR, Solit DB (2010). PIK3CA mutation uncouples tumor growth and cyclin D1 regulation from MEK/ERK and mutant KRAS signaling. Cancer Res..

[9] Wee S, Jagani Z, Xiang KX, Loo A, Dorsch M, Yao YM (2009). PI3K pathway activation mediates resistance to MEK inhibitors in KRAS mutant cancers. Cancer Res..

[10] Temraz S, Mukherji D, Shamseddine A (2015). Dual Inhibition of MEK and PI3K Pathway in KRAS and BRAF Mutated Colorectal Cancers. Int. J. Mol. Sci..

[11] Curtin JC, Lorenzi MV (2010). Drug discovery approaches to target Wnt signaling in cancer stem cells. Oncotarget.

[12] Huang SM, Mishina YM, Liu S, Cheung A, Stegmeier F, Michaud GA (2009). Tankyrase inhibition stabilizes axin and antagonizes Wnt signalling. Nature.

[13] Hong SW, Moon JH, Kim JS, Shin JS, Jung KA, Lee WK (2014). p34 is a novel regulator of the oncogenic behavior of NEDD4-1 and PTEN. Cell Death Differ..

[14] Tentler JJ, Nallapareddy S, Tan AC, Spreafico A, Pitts TM, Morelli MP (2010). Identification of predictive markers of response to the MEK1/2 inhibitor selumetinib (AZD6244) in K-ras-mutated colorectal cancer. Mol. Cancer Ther..

[15] Jeong, W.-J., Ro, E. J. & Choi, K.-Y. Interaction between Wnt/β-catenin and RAS-ERK pathways and an anti-cancer strategy via degradations of β-catenin and RAS by targeting the Wnt/β-catenin pathway. *NPJ Precis. Oncol.* **2**, 5 (2018). 10.1038/s41698-018-0049-y PMC5871897 29872723

[16] Orford K, Crockett C, Jensen JP, Weissman AM, Byers SW (1997). Serine phosphorylation-regulated ubiquitination and degradation of beta-catenin. J. Biol. Chem..

[17] Solit DB, Garraway LA, Pratilas CA, Sawai A, Getz G, Basso A (2006). BRAF mutation predicts sensitivity to MEK inhibition. Nature.

[18] Ilyas M, Tomlinson IP, Rowan A, Pignatelli M, Bodmer WF (1997). Beta-catenin mutations in cell lines established from human colorectal cancers. Proc. Natl Acad. Sci. USA.

[19] Kaler P, Augenlicht L, Klampfer L (2012). Activating mutations in beta-catenin in colon cancer cells alter their interaction with macrophages; the role of snail. PLoS One.

[20] Morin PJ (1999). beta-catenin signaling and cancer. Bioessays.

