# Supplementary material for: Targeting β-catenin overcomes MEK inhibition resistance in colon cancer with KRAS and PIK3CA mutations
Source: Br J Cancer. 2019 Apr 4;120(9):941–51. doi: 10.1038/s41416-019-0434-5 (PMC6734664; doi:10.1038/s41416-019-0434-5)
Supplement: Supplementary file 1 — Supplementary Figure and Table legend [file 41416_2019_434_MOESM1_ESM.docx]

**Supplementary Figure and Table legend**

**Supplementary Figure S1**. Downregulation of β-catenin by MEK inhibitor in *PIK3CA* wild-type cells is dependent on protein level but not mRNA level.

SW620 and DLD-1 cells were treated with 1 μM MEK inhibitor, and then β-catenin mRNA expression was analyzed by RT-PCR. GAPDH was used as a loading control.

**Supplementary Figure S2**. The expression of β-catenin was decreased in MEK inhibitor treated SW620 cells. We treated MEK inhibitor in SW620 and DLD-1 cell lines. After 24hrs, we washed, fixed, and stained with anti- β-catenin antibody. DAPI stained nucleus.

**Supplementary Figure S3**. MEK inhibitor was decreased WNT signaling in SW620 cell line. TOP/FOP flash luciferase reporter were transfected into SW620 (left panel) and DLD-1 (right panel), and incubated with MEK inhibitor (1µM), followed by 24hrs. The TOP/FOPflash luciferase activity was measured. Statistical comparative analyses between TOPflash and FOPflash in control or drug-treated cell lines: **P < 0.05; **P < 0.01*

**Supplementary Figure S4**. The percentage of apoptotic cells was determined by annexin V/PI staining. MEK inhibitor –treated and control cells were washed with PBS, stained with annexin V and PI staining solution. We analyzed early and late apoptotic population by flow cytometry. **P<0.05, **P<0.01*

**Supplementary Figure S5**. Expression of β-catenin was analyzed by RT-PCR in sc and β-catenin siRNA transfected cells. HCT8 (PIK3CA mt) cells were transfected with β-catenin siRNA. After 48hrs, we harvested and analyzed β-catenin expression by RT-PCR. GAPDH was a loading control.

**Supplementary Figure S6.** Combinational treatment of MEK inhibitor and NVP-TNKS656 was decreased WNT signaling in DLD-1 isogenic cell lines (#353). TOP/FOP flash luciferase reporter were transfected into cells, and incubated with single or both MEK inhibitor (1µM) and NVP-TNKS656 (5µM) followed by 24hrs. The TOP/FOPflash luciferase activity was measured. Statistical comparative analyses between TOPflash and FOPflash in control or drug-treated cell lines: **P < 0.05; **P < 0.01*

**Supplementary Figure S7**. β-Catenin genotype determines the sensitivity to combinatorial treatment.

HCT116 parent and wt KO (mut/-) cells did not respond to combined treatment with NVP-TNKS656 and MEK inhibitor (GSK112012). Combined treatment (Tankyrase inhibitor, NVP-TNKS656 and the MEK inhibitor GSK112012) induced cell death in HCT116 mut KO (wt/-) cells. **P<0.05, **P<0.01*

**Supplementary Figure S8**. Tumor size was decreased by combinational treatment, such as GSK112012 and NVP-TNKS656. We administrated GSK112012 (3mpk), NVP-TNKS656 (100mpk), or GSK112012 and NVP-TNKS656 on daily and measured tumor size every 3 days in PDX model. (A) Combinational effects were shown that tumor growth was inhibited in GSK112012 and NVP-TNKS656 treated groups. **P<0.05, **P<0.01* (B) We provided mouse images for vehicle, GSK112012, NVP-TNKS6556, and combinational treatment.

**Supplementary Table S1**. The lists were shown genotype and drug response.

**Supplementary Table S2**. List of differential expressed protein in MEK inhibitor treated DLD-1 and SW620

List of differential expressed protein in MEK inhibitor treated DLD-1 and SW620
