# Supplementary figures and images for: Targeting β-catenin overcomes MEK inhibition resistance in colon cancer with KRAS and PIK3CA mutations
Source: Br J Cancer. 2019 Apr 4;120(9):941–51. doi: 10.1038/s41416-019-0434-5 (PMC6734664; doi:10.1038/s41416-019-0434-5)

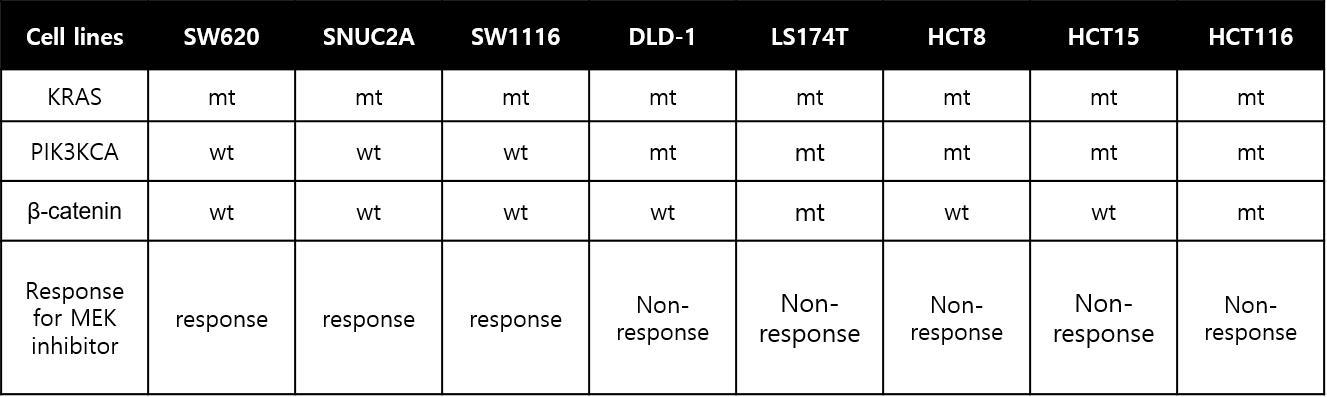


**Supplementary Table S1**

Supplement: Supplementary file 10 — Supplementary Table S1 [file 41416_2019_434_MOESM10_ESM.docx]
